# Risk of falls in the older adult at Thai Binh Medical University Hospital and its related factors in 2024

**DOI:** 10.3389/fpubh.2025.1609745

**Published:** 2025-08-04

**Authors:** Nguyen The Diep, Tien Van Nguyen, Bui Thi Minh Phuong, Nguyen Duc Thanh, Duc-Cuong Le, Nguyen Trong Duynh, Nham Tien Quynh

**Affiliations:** ^1^Department of Traumatology, Thai Binh University of Medicine and Pharmacy, Thai Binh, Vietnam; ^2^Department of Healthcare Organization and Management, Faculty of Public Health, Thai Binh University of Medicine and Pharmacy, Thai Binh, Vietnam; ^3^Department of Biochemistry, Thai Binh University of Medicine and Pharmacy, Thai Binh, Vietnam; ^4^Department of Epidemiology, Faculty of Public Health, Thai Binh University of Medicine and Pharmacy, Thai Binh, Vietnam; ^5^Department of Nursing for Adults and the Elderly, Faculty of Nursing, Thai Binh University of Medicine and Pharmacy, Thai Binh, Vietnam

**Keywords:** risk of falling, older adult, related factors, decision tree, Vietnam

## Abstract

**Background:**

Falls represent a significant health threat to the older adult, often leading to severe disability and a decline in functional independence. Effective strategies for early prediction and detection are therefore essential to ensure the health and safety of older adults. This study aims to evaluate the proportion of fall risk and some factors related to fall risk of the older adult living at the Examination Department, Thai Binh University of Medicine Hospital.

**Methods:**

A cross-sectional study was conducted on 404 older adults who presented as outpatients at Thai Binh Medical University Hospital between October 2023 and June 2024. Data were collected via direct interviews using the Vietnamese-translated STEADI-CDC Fall Risk Questionnaire (FRQ). A decision tree model was employed to identify the most significant predictive factors for fall risk.

**Results:**

Among the 404 older adults participating in the study, the risk of falling was 19.6%. The average FRQ score was 11.0 ± 2.7 points. Similarly, older adults with osteoarthritis had a risk of falling of 18.3%, while in the group without osteoarthritis, it was 5.6%. Decision tree analysis revealed two important factors that increased the risk of falling: a history of previous falls and osteoarthritis status.

**Conclusion:**

A history of previous falls and the presence of bone and joint disease are critical predictive factors for fall risk in this older adult outpatient population. These findings suggest a simplified, two-question screening approach could be effectively implemented in clinical practice to identify high-risk individuals for targeted preventive interventions.

## 1 Introduction

A fall is an event that occurs when a person accidentally falls to the ground or another lower area or sometimes when a body part hits another object, causing the person to fall. According to a WHO report, it is estimated that each year in Vietnam, about 1.5–1.9 million older adults fall, and 5% of them are hospitalized because of injuries (1). The rate of older adult falls in cross-sectional studies in Vietnam varies significantly. In the study of Giau et al. (2), the rate of older adult falls was 18.3%. Research by Ngoc and Duyen (3) showed that the rate of falls in older people with knee osteoarthritis was 23.3%, while older adult without knee osteoarthritis was 16.7%; research by Tang Thi Hao showed that the rate of falls in older people is 35.3% and the high risk of falls is 47.8% (4); Research by Huyen et al. showed that the risk of falling in older adult inpatients is high at 64.2% and a longitudinal study showed that the fall rate in outpatient older adult was 35.5% (5, 6).

Many factors increase the risk of falls in older people. Subjective factors may include fear of falling or self-reported unsteadiness, while objective factors encompass a range of elements such as a history of previous falls, polypharmacy (the use of multiple medications), visual impairments, cognitive decline, muscle weakness, gait and balance disorders, and environmental hazards. Among them, frailty syndrome is the leading risk factor, followed by visual impairment, hypotension, and polypharmacy. It may be better to state as “fractures” instead of “broken bones” due to falls (6). According to the Fall Prevention Guidelines, it is urgent to develop a standardized approach to assessing individual fall risk to stratify these risks before applying interventions. It is crucial to develop a standardized fall risk assessment to stratify individuals, allowing for interventions appropriate to their specific risk level; in particular, this assessment should quickly differentiate between those at high and low fall risk (7). Fall risk assessment tools have been proven to reduce falls and injuries caused by falls in older people in the community (8).

Decision trees offer several advantages for analyzing fall risk factors. They are non-parametric models that can handle complex interactions between variables and identify subgroups with varying risk levels, which is particularly useful given the multifactorial nature of falls. Their graphical output is intuitive and easily translatable into clinical screening questions or pathways. While accuracy can vary depending on the dataset and specific algorithm used, studies have shown decision trees to be effective in predicting falls with reasonable accuracy, sometimes comparable or even superior to traditional. Decision tree methods have been applied in many fields for prediction and classification with accurate results and comprehensible interpretation. Decision tree analysis is based on the values of independent variables and divides cases into subgroups. Moreover, the decision tree model’s results can also predict factors that increase the risk of falls in the older adult in the future and provide screening standards to immediately classify the risk of high and low falls, thereby helping to devise immediately effective strategies to reduce the older adult’s fall rate. A recent report by Deschamps et al. used decision tree analysis to predict fall risk for community-dwelling older adult with no history of falls (9). Moreover, investigating fall risk within a hospital outpatient setting is particularly important. This environment serves as a primary point of contact with the healthcare system for a large volume of older adults, many of whom have chronic conditions that elevate their fall risk. Assessing patients in this setting provides a critical opportunity for early detection and the implementation of preventive strategies before an acute fall-related event leads to hospitalization. Thai Binh Medical University Hospital is a major provincial referral center, serving a diverse population from both urban and rural areas. Its role as a key healthcare provider makes it a highly relevant site for investigating health issues affecting the region’s older adult population.

To our knowledge, fall risk factors in Vietnam can differ from other countries due to distinct environmental hazards in homes and public spaces, such as traditional building materials and crowded, uneven walkways. Socio-cultural aspects, including a rapidly aging population within multi-generational households, economic constraints impacting safety modifications, and varying levels of fall prevention awareness, also contribute to these differences. Furthermore, specific healthcare system characteristics, such as patterns of chronic disease management, medication practices, identified inpatient risks like nocturia, and less comprehensive fall data collection, distinguish Vietnam’s fall risk profile. Some previous studies in Vietnam showed that some risk factors for falls for inpatients and outpatients, but they have yet to use decision tree analysis techniques. Therefore, the present study aimed to evaluate the fall status of older adult patients in the Department of Examination and to present a simple, accurate fall prediction method by performing decision tree analysis to identify factors related to fall risk in the older adult.

## 2 Methods

### 2.1 Study design and sampling

A cross-sectional study was conducted on outpatients aged 60 years and older who were examined at the Department of Examination, Thai Binh Medical University Hospital, between October 2023 and June 2024. Participants were required to have normal cognition, a permanent residence, have lived in the research area for at least 12 months, voluntarily participate in the research and possessed adequate Vietnamese language communication skills to understand and respond to the questionnaire. Patients with a documented history of moderate to severe dementia or those who were unable to coherently answer basic orientation questions (e.g., name, age, current location, reason for visit) during the initial interaction were excluded.

The sample size was calculated using the formula for estimating a single proportion:


$$n_{1}=\frac{{{Z^{2}}_{(1-}}_{a/2)}p(1-p)}{d^{2}}$$

Based on a previous study in Vietnam, which reported a fall rate (p) of 18.3% (2) with a 95% confidence level (*Z* = 1.96) and a margin of error (d) of 4%, the minimum required sample size was 363. To account for potential incomplete responses, we aimed for a larger sample. A consecutive sampling method was employed, where all eligible patients visiting the department during the study period were invited to participate. After excluding incomplete records, a final sample of 404 participants was included in the analysis.

Patients with acute critical illness, advanced disease, and severe dementia were excluded. 404 patients of adequate data were obtained after excluding incomplete data. The patients were divided into a fall and a non-fall group based on whether they had at least one fall in the previous year.

### 2.2 Tools and methods of data collection

#### 2.2.1 Data collection tool

Data were collected by directly interviewing older people using a pre-designed questionnaire. After measuring height, weight, and blood pressure, the older adult were invited to the interview at the Examination Department, Thai Binh University of Medicine and Pharmacy Hospital. The purpose of interviewing the older adult was to collect information about their general characteristics, living habits, medication use, history of illness, previous falls, and current fall risk.

#### 2.2.2 The method of data collection

The study’s data collection technique is interviewing according to a set of prepared questions. Investigators have experience in community surveys and are fully trained in tools and methods of collecting information.

The interview aimed to collect data on: General Characteristics: Age, gender, religion, education level, occupation, economic status, living situation; Living Habits: Smoking history, alcohol consumption, use of stimulants, exercise participation, involvement in recreational and social activities; Health Status and History: History of previous falls within the past year, specific comorbidities (hypertension, diabetes, heart-related diseases, digestive diseases, bone and joint disease, urinary diseases), and medication use (though polypharmacy was not a direct focus of the final decision tree model presented here, general illness history was noted); Fall Risk Assessment: Responses to the 12 items of the STEADI-CDC Fall Risk Questionnaire (FRQ) to determine the FRQ score and fall risk status.”

### 2.3 Evaluation standards

### 2.4 Risk of falls in the older adult

The FRQ (Fall Risk Questionnaire), the fall risk assessment toolkit of STEADI-CDC-2017 USA, has been translated into Vietnamese. The older adult’s fall risk assessment scale includes 12 questions with a total score of 14 (Questions C1 and C2 have two score levels, 2 and 0). The scale is highly reliable with Cronbach’s alpha coefficient of 0.88 (4). Older adults are considered at risk of falling when the total score is four points or more and not at risk of falling when the total score is less than four points.

### 2.5 Data processing

All collected data were double-entered using EpiData 3.1 software. After completing the data entry, the data were cleaned by comparing the two entries and correcting any errors from the data entry process. After collection, the data were synthesized and processed using IBM SPSS Statistics for Windows (Version 27.0).

- Descriptive statistics: Applied to describe data about research subjects’ information according to fall risk status: Number, rate (%); Mean (X) ± Standard deviation (SD) for variables following normal distribution; Median (range) for variables that do not follow a normal distribution.
- Inferential statistics: Use *χ*^2^ test, Fisher’s exact to compare proportions; t-student test, Mann–Whitney-U to compare averages. Univariate and multivariate logistic regression analysis to learn some factors related to the risk of falls in the older adult (statistically significant with 95%CI and *p < 0.05). Using decision tree modeling to predict related factors that increase the risk of falls in the older adult.

### 2.6 Research ethics

The Ethics Council of Thai Binh University of Medicine and Pharmacy approved the current study according to Decision No 0224/IRB on July 22, 2024. The study team explained the purpose and content of the research to subjects before they answered the questions, and they had the right to refuse to answer if they did not want to. The participation of all subjects in the study is entirely voluntary, and all information about the research subjects is kept confidential. All information of research participants is encrypted and confidential. The data and information collected in the reports are committed to being used for research purposes, not other purposes. The research results and proposed proposals will be used to improve health care for patients coming for examination and treatment at Thai Binh Medical University Hospital.

## 3 Results

### 3.1 General characteristics of research subjects

In Table 1, 404 older people participated in the study, 19.6% of the study subjects were at risk of falling and the average fall risk score was high (11.0 ± 2.7) (Table 2).

**Table 1:** Rate of fall risk of study subjects.

Risk of falling	*n*	%
Yes	79	19.6
No	325	80.4
FRQ score ( $\bar{X}$ ± SD)	11.0 ± 2.7	

FRQ, Fall Risk Questionnaire; SD, standard deviation.

**Table 2:** Demographic characteristics according to fall risk status in the older adult.

Characteristic		Yes (*n* = 79)		No (*n* = 325)		*p*-value
		n	%	n	%	
Age group	60–70	36	15.3	200	84.7	<0.05
	70–80	31	23.5	101	76.5	
	≥ 80	12	33.2	24	66.8	
Religion	No	65	17.2	312	82.8	<0.001
	Yes	14	51.9	13	48.1	
Academic level	≤ Primary school	15	27.8	39	72.2	<0.05
	Secondary school	28	14.4	166	85.6	
	High school	29	27.9	75	72.1	
	≥ College	7	13.5	45	86.5	
Occupation	Manual labor	45	18.8	195	81.2	>0.05
	Mental work	34	20.7	130	79.3	
Economic status	Dependent	12	25.5	35	74.5	>0.05
	Free	67	18.8	290	81.2	
Living situation	Alone	45	18.7	196	81.3	>0.05
	Live with family	34	20.9	129	79.1	

Distribution of demographic characteristics of the older adult according to fall risk showed that: The older the age group, the higher the fall risk rate. In the group of older adult people ≥ 80 years old, the rate at risk of falling is 33.2%, higher than other age groups. In addition, the difference in the fall risk rate of older adult people across religious groups and education levels is statistically significant (*p*<0.05).

Results in Table 3 showed that the proportion of older adult people who smoke, drink alcohol, and use stimulants in the group at risk of falling is higher than the group without the risk of falling.Older adult people’s exercise habits according to the risk of falling (Table 4) show that, within the group of older adult people who did not participate in recreational activities, the proportion at risk of falling (47.1%) was significantly higher than the proportion at risk of falling (18.3%) among those who did participate in recreational activities (*p* < 0.05).

**Table 3:** Distribution of eating habits of the older adult according to fall risk.

Characteristic		Yes (*n* = 79)		No (*n* = 325)		*p*-value
		n	%	n	%	
Smoking history	Yes	13	25.5	38	74.5	>0.05
	No	66	18.7	287	81.3	
History of drinking alcohol	Yes	20	23.3	66	76.7	>0.05
	No	59	18.6	259	81.4	
Using stimulants	Yes	33	19.9	133	80.1	>0.05
	No	46	19.3	192	80.7	

**Table 4:** Distribution of movement habits of the older adult according to fall risk.

Characteristic		Yes (*n* = 79)		No (*n* = 325)		*p*-value
		n	%	n	%	
Do exercise	Yes	64	18.3	286	81.7	>0.05
	No	15	27.8	39	72.2	
Participate in recreational activities	Yes	71	18.3	316	81.7	<0.05
	No	8	47.1	9	52.9	
Participate in social activities	Yes	52	18.9	223	81.1	>0.05
	No	27	20.9	102	79.1	

In Table 5, except for urinary tract diseases, the proportion of older adult in the remaining five diseases in the group with a risk of falling is all statistically significantly higher than the group without a risk of falling. Older adult people with a history of previous falls have a higher risk of falling than other older adults (*p*<0.05).

**Table 5:** Distribution of pathological characteristics according to the risk of falling.

Characteristics		Yes (*n* = 79)		No (*n* = 325)		*p*-value
		n	%	n	%	
Including diseases	Hypertension	32	26.2	90	73.8	<0.05
	Diabetes	34	25.4	100	74.6	<0.05
	Heart-related diseases	21	36.2	37	63.8	<0.01
	Digestive disease	10	34.5	19	65.5	<0.05
	Bone and joint disease	29	32.6	60	67.4	<0.05
	Urinary diseases	5	26.3	14	73.7	>0.05
Have fallen before	Yes	51	77.3	15	22.7	<0.05
	No	28	8.3	310	91.7	

### 3.2 Some factors related to the risk of falls in the older adult

The results of the multivariable regression model presented in Table 6 showed that older adults who follow a religion, have an education level of primary school or less, have comorbidities, and regularly participate in entertainment activities are factors. Related to the risk of falls in the older adult participating in the study (*p* < 0.05).

**Table 6:** Multi-variable logistics analysis of factors related to the risk of falling.

Independent variables	b	SE	Wald	*p*-value	OR (95% CI)
Religion					
No	Ref.				
Yes	1.3	0.7	3.9	<0.05	3.6 (1.0, 12.8)
Academic level					
College or higher	Ref.				
Junior high school	1.3	0.9	2.2	>0/05	3.6 (0.7, 18.7)
High school	1.1	0.7	2.2	>0.05	3.0 (0.7, 12.8)
Primary school and below	1.9	0.8	6.5	<0.05	7.0 (1.6, 31.2)
History of previous falls					
No	Ref.				
Yes	2.1	0.2	79.3	<0.0001	8.5 (5.3, 15.5)
Including diseases					
No	Ref.				
Yes	1.6	0.4	13.5	<0.0001	4.7 (2.1, 10.7)
Participate in recreational activities					
No	Ref.				
Yes	−2.0	0.7	8.1	<0.01	0.1 (0.04, 0.5)

**Figure 1:**
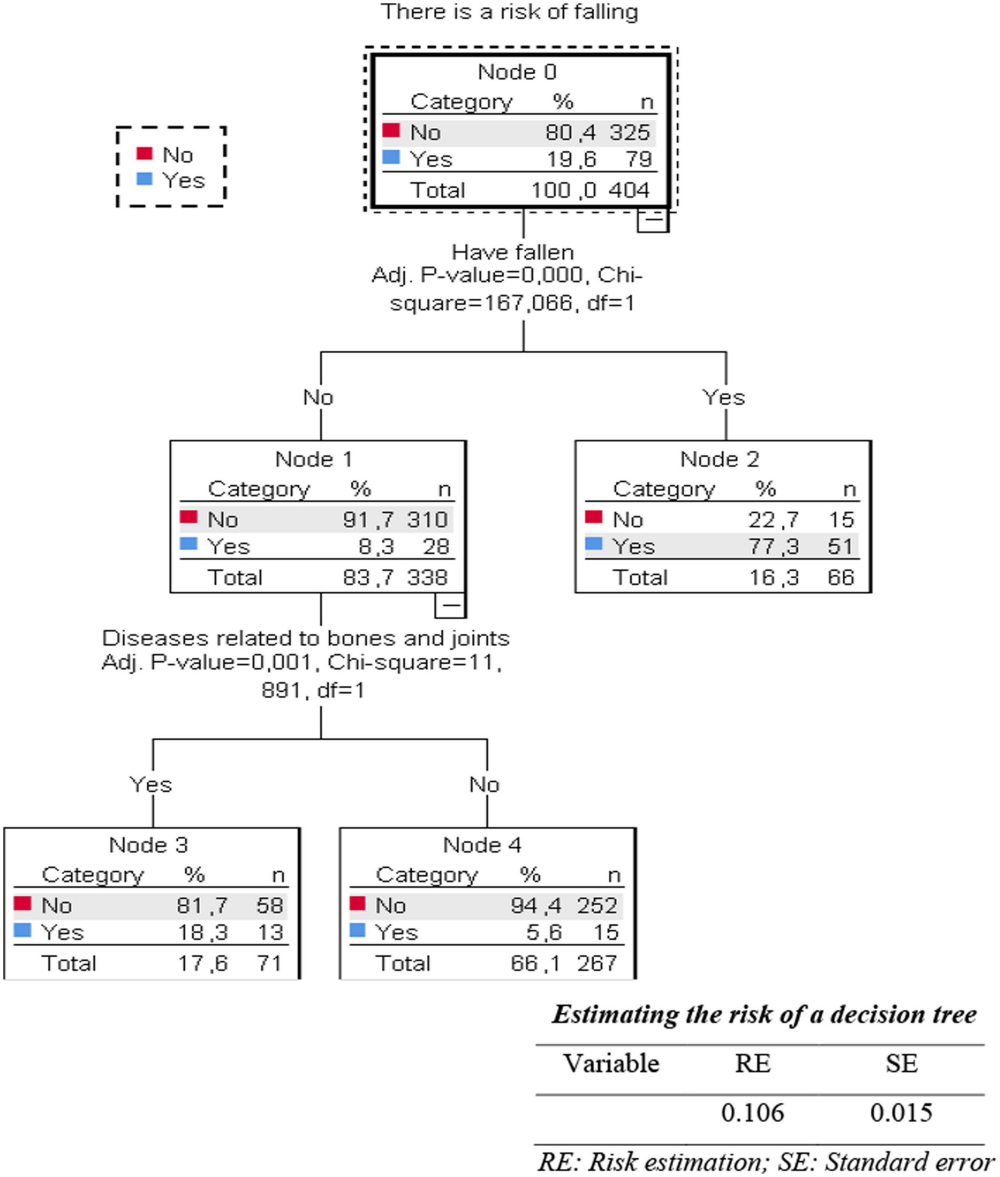
Decision tree for factors related to fall risk in the older adult.

In Figure 1, the results of the decision tree analysis to predict factors related to the risk of falling in the older adult in the current study were determined to include two factors: history of falling and bone and joint disease (*p* < 0.05).

## 4 Discussion

Our study was conducted to identify some factors related to the risk of falls in 404 older adult people, with 79 older adult people at risk of falling (19.5%). Statistically significant differences (*p* < 0.05) were observed between the group at risk of falling and the group not at risk of falling concerning age, religion, education level, participation in recreational activities, history of previous falls, and the presence of certain accompanying diseases such as hypertension, diabetes, cardiovascular disease, and bone and joint disease. The difference between the two groups of older adult people at risk of falling and without risk of falling was statistically significant (*p* < 0.05). This result is similar to the research results of Nguyen Thi My Dung and colleagues (10). Pathological conditions may affect posture, balance and gait in the older adult, thereby increasing the risk of falls. Not only that, during the investigation, the research team found that older adult people who had fallen before thought they were more likely to fall due to both physiological and psychological changes.

In this study, we used a decision tree model to predict related factors that increase the risk of falls in older people. The decision tree model showed two factors that could be used to assess risk, including fall screening for older adults in the community: the history of previous falls and the presence of bone and joint disease (*p* < 0.05). Our decision tree model identified a history of previous falls and bone and joint disease as key predictors. While Giau et al. and Ngoc et al. highlighted associations with hypertension and knee osteoarthritis, respectively, using traditional analyses, our study contributes a simplified predictive model derived from decision tree analysis specifically for an outpatient setting in Thai Binh. The strong emphasis on previous falls aligns with findings by Dung et al., but our use of a decision tree offers a more direct screening pathway. On the other hand, bone and joint diseases such as osteoporosis and osteoarthritis have been proven to be factors that increase the risk of falls in older adults in the community, both at home and abroad (2, 6, 10). The natural aging process is accompanied by bone and joint diseases, causing the calcium content in bones in older adult patients to decrease significantly, accompanied by a decline in muscle mass, strength, and musculoskeletal system function.

Furthermore, older adults with a history of previous falls had a fall risk rate of 77.3%, while in the group without a prior history of falls, this rate was 8.3%. Similarly, older adults with bone and joint disease have a fall risk rate of 32.6%, while in the group without bone and joint disease, it is 5.6%. History of falls (or having fallen before) is related to the risk of falls in older adults in the community and has been presented in several previous studies. Barak, when comparing older adult patients who fell within six months before the time of the study, found that 57.0% of these cases were unable to walk at an average speed as before the fall with other patients with short steps and high amplitude of bilateral fluctuations during each step (11). Compared with older adults who did not fall, the variability of kinematic measurements in the group of older adults who fell was significantly increased (*p* < 0.05) (11). These changes reflect mobility limitations that increase the risk of (subsequent) falls. In addition, older adults with a history of falls are also associated with symptoms of depression and anxiety, which are all factors that increase the risk of falls.

Our findings can only be applied to those older people who can ambulate independently in the community. Although self-reporting of fall events has been criticized due to the tendency to under-report actual fall events, the clinical value of this retrospective self-reported method in reflecting the exact conditions of falls is demonstrated because it is still widely used in fall risk studies (12, 13). However, a 12-month recall is still better than a recall for falls in the previous 3 or 6 months. Screening for falls and fall risk is aimed at preventing or reducing fall risk, persons screened into a high-risk group warrant further evaluation. We believe that self-reporting of recall of fall events was useful in fall detection for preventing or reducing fall risk among older adult people.

Our study has limitations. As participants were interviewed at a single point in time during their outpatient clinic visit, this cross-sectional design limits our ability to conduct a comprehensive, long-term assessment or establish causality. Additionally, self-reporting of fall history introduces potential recall bias, although we used a 12-month recall period, which is considered more reliable than shorter intervals for capturing fall events. Second, the prevalence of recurrent fallers (those experiencing multiple falls within the year) in our sample was low. Consequently, an in-depth analysis of risk factors specifically for this subgroup could not be robustly performed, and they were grouped with single-fallers in the “history of previous falls” category. Third, the participants were healthy older adults, so the algorithms developed in this study may not be able to predict falls in high-risk groups. Furthermore, cultural differences and healthcare support structures specific to Vietnam may influence the generalizability of these conclusions. For example, strong family support systems common in Vietnam might mitigate some fall risks for older adult individuals living with relatives, a factor that could differ in societies with more independent living arrangements for the older adult. Access to and utilization of primary healthcare services, community-based fall prevention programs, and public awareness campaigns regarding fall risks also vary significantly across countries and can impact both the prevalence of risk factors and the effectiveness of predictive models.

## 5 Conclusion

Our study confirms that a history of previous falls and the presence of bone and joint disease are the most critical predictors of fall risk among older adult outpatients in Vietnam. The analysis using a decision tree model showed that a history of a recent fall and the status of bone and joint disease were predictive factors that could be used to screen for the risk of falling in the older adult who came for outpatient examination and treatment at the Examination Department, Thai Binh Medical University Hospital. Our findings provide suggestions for nursing departments to identify high-risk patients and to take preventive measures when they are admitted to the hospital. A simple two-question screening could be administered by nursing staff or during patient intake: (1) “Have you fallen in the past year?” and (2) “Do you have a history of significant bone or joint problems like osteoarthritis or osteoporosis?” Affirmative answers would flag the patient for a more comprehensive fall risk assessment using the full FRQ or referral for further evaluation and targeted interventions, streamlining the process of identifying high-risk individuals efficiently.

## Data Availability

The raw data supporting the conclusions of this article will be made available by the authors, without undue reservation.
